# Supplementary material for: A cross-sectional analysis of podiatrist-initiated review processes after issuing prescribed foot orthoses
Source: PLoS One. 2022 Oct 31;17(10):e0276716. doi: 10.1371/journal.pone.0276716 (PMC9621403; doi:10.1371/journal.pone.0276716)
Supplement: S1 File — (PDF) [file pone.0276716.s001.pdf]

Charles Sturt University

Issuing and Reviewing Orthotic Devices in Podiatric Practice (draft)

Participant Information

You are invited to participate in a project titled:
ISSUING ORTHOTIC DEVICES AND REVIEWING THEIR EFFICACY: AN INVESTIGATION OF COMMON PROCEDURES AND DEVELOPMENT OF STANDARDISED GUIDELINES (PHASE 1)

The study is being conducted by Luke Donnan, Emma Baker and Anna Horn from the School of Community Health at Charles Sturt University.

Before you decide whether or not you wish to participate in this study, it is important for you to understand why the research is being done and what it will involve. Please take the time to read the following information carefully and discuss it with others if you wish.

What is the purpose of the study?
This study seeks to identify common practices when issuing orthotics, identify timeframes for follow-up after an orthotic device is provided to a patient, and examine the potential influence of orthotic provision procedures and patient outcomes. We are currently conducting Phase 1 of the study, which is focussing on how orthotics are issued by practising podiatrists.

Why am I being invited to participate in this study?
You have been identified as a qualified podiatrist, and your insights regarding the issuing of a pair of orthotics are critical to us to enable the development of standardised clinical guidelines relating to the issuing of orthotics.

What does this study involve?
Your participation in the study will involve completion of this online survey in which you will be asked to explain the processes and procedures you use when issuing a pair of orthotics. The survey is expected to take less than 10 minutes to complete.

Are there any risks and benefits to me taking part in this study?
There will be no foreseeable risks or direct benefits to you from participating in this research. Your decision to participate or not will have no direct consequences for you.

How is this study being paid for?
The study is currently unfunded, and Phase 1 of the study will not require funding. External funding will be sought for later phases of the study.

Will taking part in this study cost me anything, and will it be paid for?
The survey will not cost you anything to complete. No reimbursement or payments will be provided.

What if I don't want to take part in this study?
Participation in this research is entirely your choice. Whether you decide to participate or not is your decision, and you will not be disadvantaged either way.

What if I participate and want to withdraw later?
If you start completing the survey, you can cease your involvement at any time or choose not to answer any of the questions.

How will my confidentiality be protected?
All surveys will be anonymous, and the researchers will not be aware of who has or has not completed the surveys. The information collected from the surveys will be collated, meaning no individual survey responses will be reported, further protecting the confidentiality of participants.

What will happen to the information that I give you?
If you choose to participate, your results will contribute to a larger set of data that will be analysed to identify the most common procedures used when issuing orthotic devices. Following analysis, we plan to develop at least two sets of guidelines to be tested within the Charles Sturt University podiatry clinic.

If you have a particular question about the findings of our study, you are invited to put this question in an email to Luke Donnan at ldonnan@csu.edu.au.

What should I do if I want to discuss this study further before I decide?
If you would like further information, please contact Luke Donnan at ldonnan@csu.edu.au or on (02)60519258.

Who should I contact if I have concerns about the conduct of this study?
Charles Sturt University's Human Research Ethics Committee has approved this project (Protocol number xxxxx). If you have any complaints or reservations about the ethical conduct of this project, you may contact the Committee through the Ethics and Compliance Unit via the following contact details:

The Governance Officer
Human Research Ethics Committee
Ethics and Compliance Unit
Locked Bag 588
Wagga Wagga NSW 2678
Phone: (02) 6933 4628
Email: ethics@csu.edu.au

Any issues you raise will be treated in confidence and investigated fully, and you will be informed of the outcome.

Thank you for considering this invitation.

Please print this information if you wish to keep a record for future reference.

Charles Sturt University

Issuing and Reviewing Orthotic Devices in Podiatric Practice (draft)

Consent

By completing this online survey...

- You agree to participate in the above research project and give your consent freely.
- You understand that the project will be conducted as described in the Participant Information, a copy of which you have been provided with and have retained.
- You have had an opportunity to ask questions about the project and to have them answered.
- You understand you can withdraw from the project at any time and do not have to give any reason for withdrawing.
- You understand that by completing and submitting this survey you are consenting to the above terms.

If you agree and give your consent, please click 'Next' to continue to the survey questions.

Charles Sturt University

Issuing and Reviewing Orthotic Devices in Podiatric Practice (draft)

Education and Experience

1. How long have you been practising as a podiatrist?

- ◯ Less than 1 year
- ◯ 1 - 5 years
- ◯ 6 - 10 years
- ◯ 11 - 15 years
- ◯ More than 15 years

2. From which university did you graduate?

Charles Sturt University

Prescriptions

3. On average, how many pairs of orthotics would you prescribe in a standard week?

- ○ None - I don't prescribe orthotics
- ○ Less than 1 pair
- ○ 1 - 3 pairs
- ○ 4 - 6 pairs
- ○ 7 - 9 pairs
- ○ 10 or more pairs

Charles Sturt University

Issuing and Reviewing Orthotic Devices in Podiatric Practice (draft)

Prescriptions

4. For what proportion of patients do you use the following shell materials?

	More than 50%	Less than 50%	None / Rarely
EVA	◯	◯	◯
Polypropylene	◯	◯	◯
Carbon fibre	◯	◯	◯

Other (please specify)

Charles Sturt University

Issuing and Reviewing Orthotic Devices in Podiatric Practice (draft)

Orthotic Fitting

5. How often do you use the following methods to confirm the orthotic device fits the patient's foot and is achieving the intended functional change?

	Always	More than 50% of patients	Less than 50% of patients	Rarely / Never
Measure length of device in relation to metatarsal heads	○	○	○	○
Measure width of device in relation to foot	○	○	○	○
Assess congruence of arch	○	○	○	○
Achieve neutral calcaneal stance position (NCSP)	○	○	○	○
Moving away from resting calcaneal stance position (RCSP)	○	○	○	○
Jack’s test result on and off the orthotic device	○	○	○	○
Gait assessment	○	○	○	○

6. Please indicate any other methods you use to confirm fit and function:

Charles Sturt University

Issuing and Reviewing Orthotic Devices in Podiatric Practice (draft)

Patient Education

7. For what proportion of patients do you provide education when they are receiving a new orthotic?

All patients	More than 50%	Less than 50%	None
○	○	○	○

Charles Sturt University

Issuing and Reviewing Orthotic Devices in Podiatric Practice (draft)

Patient Education

8. Without considering the symptoms being treated, how long do you suggest it will take most patients to become comfortable in new orthotic devices (i.e. wearing them in)?

- ◯ Comfort should be immediate
- ◯ Less than 1 week
- ◯ 1 week
- ◯ 2 weeks
- ◯ More than 2 weeks
- ◯ Other (please specify)

9. Do you discuss the potential for new symptoms (e.g. stiffness or mild discomfort) to develop during the wearing-in process?

- ◯ Yes
- ◯ Sometimes
- ◯ No

Charles Sturt University

Issuing and Reviewing Orthotic Devices in Podiatric Practice (draft)

Patient Education

10. What sites do you discuss as having the potential to become symptomatic during the wearing-in process?
(Please indicate all that apply)

- [ ] Arch
- [ ] Heel
- [ ] Foot
- [ ] Ankle
- [ ] Calves
- [ ] Other (please specify)
- [ ] Knee
- [ ] Hip
- [ ] Back
- [ ] I don't mention specific sites

Issuing and Reviewing Orthotic Devices in Podiatric Practice (draft)

Patient Education

11. How often would you recommend new shoes following the issue of an orthotic device?

Always	Regularly	Rarely / Never	Only if the current shoes are worn
○	○	○	○

Charles Sturt University

Issuing and Reviewing Orthotic Devices in Podiatric Practice (draft)

Patient Education

12. How often would you recommend the following adjunct interventions to supplement the use of the orthotic devices?

	More than 50% of patients	Less than 50% of patients	Rarely / Never
Lower leg stretching or strengthening	◯	◯	◯
Upper leg stretching or strengthening	◯	◯	◯
Hip / Gluteal stretching or strengthening	◯	◯	◯
Strapping (rigid tape)	◯	◯	◯
Strapping (kinesiology tape)	◯	◯	◯
Padding	◯	◯	◯
Ice therapy	◯	◯	◯
Heat therapy	◯	◯	◯
Massage	◯	◯	◯
Shockwave therapy	◯	◯	◯
Ultrasound	◯	◯	◯

13. Please specify any other adjunct interventions you may recommend:

Charles Sturt University

Issuing and Reviewing Orthotic Devices in Podiatric Practice (draft)

Review Process

14. Do you schedule review consultations after the initial orthotic fitting?

- ○ Yes
- ○ No

Charles Sturt University

Issuing and Reviewing Orthotic Devices in Podiatric Practice (draft)

Review Process

15. How many weeks after the initial orthotic fitting would you schedule the first review consultation?

[ ]

16. Would you schedule a second review consultation?

- ○ Yes
- ○ No
- ○ Only if required

Charles Sturt University

Issuing and Reviewing Orthotic Devices in Podiatric Practice (draft)

Review Process

17. How many weeks after the first review consultation would you schedule the second review consultation?

18. Would you typically schedule a third or more review consultations?

- Yes
- No
- Only if required

Issuing and Reviewing Orthotic Devices in Podiatric Practice (draft)

Review Process

19. Please detail the standard scheduling of review consultations that follow the second review consultation:

Charles Sturt University

Issuing and Reviewing Orthotic Devices in Podiatric Practice (draft)

Review Process

20. How long do you recommend patients wait before using their new orthotic devices for exercise and/or competitive sport?

- No need to wait
- Less than one week
- 1 week
- 2 weeks
- More than 2 weeks
- Once the devices can be worn for a full day
- Once the patient finds the devices comfortable
- Other (please specify)

21. Once you and the patient are happy with the orthotic devices, how long would you recommend for a long term review?

- 6 months
- 12 months
- 24 months
- On a needs basis
- No long term review required
- Other (please specify)

22. How long would you typically recommend a patient continues wearing their orthotic devices for?

- Until the original symptoms resolve
- Until the patient grows out of the devices
- Ongoing use / Lifelong
- No time frame is specified
- Advice is case dependent (please specify):

Charles Sturt University

Issuing and Reviewing Orthotic Devices in Podiatric Practice (draft)

Review Process

23. How often do you recommend patients replace their orthotic devices? (More than one may be selected if applicable)

- [ ] 1 year
- [ ] 2 years
- [ ] 5 years
- [ ] 10 years
- [ ] If the devices break
- [ ] If the symptoms return
- [ ] They will not need replacing
- [ ] Advice is case dependent (please specify):

Charles Sturt University

Issuing and Reviewing Orthotic Devices in Podiatric Practice (draft)

Personal Reflection

24. How influential are the following on your orthotic issue and review processes?

	Highly influential	Moderately influential	Minimally or not influential
Appointment availability	◯	◯	◯
Clinic protocols / Employer preferences	◯	◯	◯
Laboratory based factors (e.g. manufacture and delivery times)	◯	◯	◯
Patient preferences	◯	◯	◯
Professional judgement	◯	◯	◯

25. Please specify any other influences on your review protocol process:
