# Supplementary material for: A cross-sectional analysis of podiatrist-initiated review processes after issuing prescribed foot orthoses
Source: PLoS One. 2022 Oct 31;17(10):e0276716. doi: 10.1371/journal.pone.0276716 (PMC9621403; doi:10.1371/journal.pone.0276716)
Supplement: S1 Table — (DOCX) [file pone.0276716.s002.docx]

**S2 Table.** **Response rate for each question analysed in this study.**

| **Question** | **All** |  | **< 1 year** |  | **1 - 5 years** |  | **6 - 10 years** |  | **11 - 15 years** |  | **> 15 years** |
| --- | --- | --- | --- | --- | --- | --- | --- | --- | --- | --- | --- |
|  | *n (%)* |  | *n (%)* |  | *n (%)* |  | *n (%)* |  | *n (%)* |  | *n (%)* |
| 1 | 238 (100) |  | 18 (100) |  | 73 (100) |  | 43 (100) |  | 35 (100) |  | 69 (100) |
| 2 | 230 (97) |  | 17 (94) |  | 71 (97) |  | 41 (95) |  | 34 (97) |  | 67 (97) |
| 3 | 233 (98) |  | 17 (94) |  | 72 (99) |  | 43 (100) |  | 34 (97) |  | 67 (97) |
| 4a | 217 (91) |  | 16 (89) |  | 70 (96) |  | 40 (93) |  | 33 (94) |  | 58 (84) |
| 4b | 218 (92) |  | 16 (89) |  | 69 (95) |  | 42 (98) |  | 33 (94) |  | 58 (84) |
| 4c | 200 (84) |  | 16 (89) |  | 61 (84) |  | 40 (93) |  | 27 (77) |  | 56 (81) |
| 14 | 215 (90) |  | 15 (83) |  | 68 (93) |  | 39 (91) |  | 32 (91) |  | 61 (88) |
| 15 | 208 (87) |  | 15 (83) |  | 66 (90) |  | 37 (86) |  | 32 (91) |  | 58 (84) |
| 16 | 210 (88) |  | 15 (83) |  | 66 (90) |  | 37 (86) |  | 32 (91) |  | 60 (87) |
| 17* | 52 (76) |  | 0 (0) |  | 15 (68) |  | 10 (91) |  | 9 (90) |  | 18 (78) |
| 21 | 193 (81) |  | 14 (78) |  | 62 (85) |  | 35 (81) |  | 28 (80) |  | 54 (78) |
| 24a | 211 (89) |  | 15 (83) |  | 66 (90) |  | 39 (91) |  | 31 (89) |  | 60 (87) |
| 24b | 211 (89) |  | 15 (83) |  | 66 (90) |  | 39 (91) |  | 31 (89) |  | 60 (87) |
| 24c | 211 (89) |  | 15 (83) |  | 66 (90) |  | 39 (91) |  | 31 (89) |  | 60 (87) |
| 24d | 210 (88) |  | 15 (83) |  | 65 (89) |  | 39 (91) |  | 31 (89) |  | 60 (87) |
| 24e | 211 (89) |  | 15 (83) |  | 67 (92) |  | 39 (91) |  | 31 (89) |  | 59 (86) |

Percentage values calculated relative to the number of responses recorded for the respective groups at question 1. *Question 17 was a contingency question from question 16. Percentages reported for question 17 have been calculated based on the number of respondents to answer ‘yes’ to question 16, as only those respondents gained access to question 17. *n* of participants answering ‘yes’ to question 16: All (*n*=68), < 1 year (*n*=2), 1-5 years (*n*=22), 6-10 years (*n*=11), 11-15 years (*n*=10), >15 years (*n*=23).
