# Supplementary material for: A cross-sectional analysis of podiatrist-initiated review processes after issuing prescribed foot orthoses
Source: PLoS One. 2022 Oct 31;17(10):e0276716. doi: 10.1371/journal.pone.0276716 (PMC9621403; doi:10.1371/journal.pone.0276716)
Supplement: S2 Table — (DOCX) [file pone.0276716.s003.docx]

**S3 Table. Proportion of different types of foot orthoses prescribed relative to the practitioner’s years of clinical experience.**

|  | **All** |  | **< 1 year** |  | **1 - 5 years** |  | **6 - 10 years** |  | **11 - 15 years** |  | **> 15 years** |
| --- | --- | --- | --- | --- | --- | --- | --- | --- | --- | --- | --- |
|  | *n (%)* |  | *n (%)* |  | *n (%)* |  | *n (%)* |  | *n (%)* |  | *n (%)* |
| *EVA* | | | | | | | | | | | |
| None / Rarely | 55 (25) |  | 5 (31) |  | 18 (26) |  | 16 (40) |  | 2 (6) |  | 14 (24) |
| Less than 50% | 87 (40) |  | 5 (31) |  | 26 (37) |  | 9 (23) |  | 17 (52) |  | 30 (52) |
| More than 50% | 75 (35) |  | 6 (38) |  | 26 (37) |  | 15 (38) |  | 14 (42) |  | 14 (24) |
| **Total** | **217** |  | **16** |  | **70** |  | **40** |  | **33** |  | **58** |
|  | | | | | | | | | | | |
| *Polypropylene* | | | | | | | | | | | |
| None / Rarely | 31 (14) |  | 2 (13) |  | 11 (16) |  | 7 (17) |  | 4 (12) |  | 7 (12) |
| Less than 50% | 80 (37) |  | 6 (38) |  | 29 (42) |  | 17 (40) |  | 14 (42) |  | 14 (24) |
| More than 50% | 107 (49) |  | 8 (50) |  | 29 (42) |  | 18 (43) |  | 15 (45) |  | 37 (64) |
| **Total** | **218** |  | **16** |  | **69** |  | **42** |  | **33** |  | **58** |
|  | | | | | | | | | | | |
| *Carbon fibre* | | | | | | | | | | | |
| None / Rarely | 164 (82) |  | 14 (88) |  | 49 (80) |  | 35 (88) |  | 22 (81) |  | 44 (79) |
| Less than 50% | 24 (12) |  | 1 (6) |  | 6 (10) |  | 5 (13) |  | 4 (15) |  | 8 (14) |
| More than 50% | 12 (6) |  | 1 (6) |  | 6 (10) |  | 0 (0) |  | 1 (4) |  | 4 (7) |
| **Total** | **200** |  | **16** |  | **61** |  | **40** |  | **27** |  | **56** |
|  |  |  |  |  |  |  |  |  |  |  |  |
| *Other (please specify)* | | | | | | | | | | | |
| 3D print PA 11 | 31 (69) |  | 0 (0) |  | 9 (60) |  | 12 (86) |  | 3 (75) |  | 7 (70) |
| Off the shelf | 7 (16) |  | 1 (50) |  | 2 (13) |  | 2 (14) |  | 0 (0) |  | 2 (20) |
| Subortholene | 2 (4) |  | 1 (50) |  | 1 (7) |  | 0 (0) |  | 0 (0) |  | 0 (0) |
| Other | 5 (11) |  | 0 (0) |  | 3 (20) |  | 0 (0) |  | 1 (25) |  | 1 (10) |
| **Total** | **45** |  | **2** |  | **15** |  | **14** |  | **4** |  | **10** |

EVA ethylene vinyl acetate, 3D three-dimensional, *n* number of respondents in each category, % percentage of respondents in each category proportional to the total number of respondents with equivalent years of practice experience.
