# Supplementary material for: A cross-sectional analysis of podiatrist-initiated review processes after issuing prescribed foot orthoses
Source: PLoS One. 2022 Oct 31;17(10):e0276716. doi: 10.1371/journal.pone.0276716 (PMC9621403; doi:10.1371/journal.pone.0276716)
Supplement: S3 Table — (DOCX) [file pone.0276716.s004.docx]

**S4 Table. Scheduling procedures adopted by respondents when performing an initial foot orthosis review consultation.**

|  | **All** |  | **<1 year** |  | **1-5 years** |  | **6-10 years** |  | **11-15 years** |  | **>15 years** |
| --- | --- | --- | --- | --- | --- | --- | --- | --- | --- | --- | --- |
|  | *n (%)* |  | *n (%)* |  | *n (%)* |  | *n (%)* |  | *n (%)* |  | *n (%)* |
| *How many weeks after the initial orthotic fitting would you schedule the first review consultation?* | | | | | | | | | | | |
| 1 week | 3 (1) |  | 0 (0) |  | 0 (0) |  | 2 (5) |  | 0 (0) |  | 1 (2) |
| 2 weeks | 69 (33) |  | 4 (27) |  | 21 (32) |  | 14 (38) |  | 11 (34) |  | 19 (33) |
| 3 weeks | 24 (12) |  | 0 (0) |  | 9 (14) |  | 5 (14) |  | 3 (9) |  | 7 (12) |
| 4 weeks | 84 (40) |  | 9 (60) |  | 28 (42) |  | 10 (27) |  | 18 (56) |  | 19 (33) |
| 5 weeks | 2 (2) |  | 0 (0) |  | 0 (0) |  | 0 (0) |  | 0 (0) |  | 2 (3) |
| 6 weeks | 20 (10) |  | 2 (13) |  | 5 (8) |  | 5 (14) |  | 0 (0) |  | 8 (14) |
| 8 weeks | 4 (2) |  | 0 (0) |  | 1 (2) |  | 1 (3) |  | 0 (0) |  | 2 (3) |
| 12 weeks | 1 (0) |  | 0 (0) |  | 1 (2) |  | 0 (0) |  | 0 (0) |  | 0 (0) |
| 14 weeks | 1 (0) |  | 0 (0) |  | 1 (2) |  | 0 (0) |  | 0 (0) |  | 0 (0) |
| **Total** | **208 (100)** |  | **15** |  | **66** |  | **37** |  | **32** |  | **58** |

*n* number of respondents in each category, % percentage of respondents in each category proportional to the total number of respondents with equivalent years of practice experience. Durations (weeks) with no responses have been excluded from this table.
