# Supplementary material for: A cross-sectional analysis of podiatrist-initiated review processes after issuing prescribed foot orthoses
Source: PLoS One. 2022 Oct 31;17(10):e0276716. doi: 10.1371/journal.pone.0276716 (PMC9621403; doi:10.1371/journal.pone.0276716)
Supplement: S4 Table — (DOCX) [file pone.0276716.s005.docx]

**S5 Table. Scheduling procedures adopted by respondents when performing a second foot orthosis review consultation.**

|  | **All** |  | **< 1 year** |  | **1 - 5 years** |  | **6 - 10 years** |  | **11 - 15 years** |  | **> 15 years** |
| --- | --- | --- | --- | --- | --- | --- | --- | --- | --- | --- | --- |
|  | *n (%)* |  | *n (%)* |  | *n (%)* |  | *n (%)* |  | *n (%)* |  | *n (%)* |
| *How many weeks after the first review consultation would you schedule the second review consultation?* | | | | | | | | | | | |
| 1 week | 1 (1) |  | 0 (0) |  | 0 (0) |  | 1 (9) |  | 0 (0) |  | 0 (0) |
| 2 weeks | 12 (18) |  | 0 (0) |  | 4 (18) |  | 1 (9) |  | 1 (10) |  | 6 (26) |
| 3 weeks | 2 (3) |  | 0 (0) |  | 0 (0) |  | 1 (9) |  | 0 (0) |  | 1 (4) |
| 4 weeks | 20 (29) |  | 0 (0) |  | 5 (23) |  | 4 (36) |  | 5 (50) |  | 6 (26) |
| 6 weeks | 9 (13) |  | 0 (0) |  | 3 (14) |  | 2 (18) |  | 2 (20) |  | 2 (9) |
| 8 weeks | 2 (3) |  | 0 (0) |  | 1 (5) |  | 0 (0) |  | 0 (0) |  | 1 (4) |
| 12 weeks | 6 (9) |  | 0 (0) |  | 2 (9) |  | 1 (9) |  | 1 (10) |  | 2 (9) |
| 16 weeks | 2 (3) |  | 0 (0) |  | 2 (9) |  | 0 (0) |  | 0 (0) |  | 0 (0) |
| 20 weeks | 1 (1) |  | 0 (0) |  | 0 (0) |  | 0 (0) |  | 0 (0) |  | 1 (4) |
| 24 weeks | 4 (6) |  | 1 (50) |  | 1 (5) |  | 1 (9) |  | 1 (10) |  | 0 (0) |
| 26 weeks | 2 (3) |  | 1 (50) |  | 1 (5) |  | 0 (0) |  | 0 (0) |  | 0 (0) |
| 52 weeks | 7 (10) |  | 0 (50) |  | 3 (14) |  | 0 (0) |  | 0 (0) |  | 4 (17) |
| **Total** | **68** |  | **2** |  | **22** |  | **11** |  | **10** |  | **23** |

*n* number of respondents in each category, % percentage of respondents in each category proportional to the total number of respondents with equivalent years of practice experience. Durations (weeks) with no responses have been excluded from this table.
