# Supplementary material for: A cross-sectional analysis of podiatrist-initiated review processes after issuing prescribed foot orthoses
Source: PLoS One. 2022 Oct 31;17(10):e0276716. doi: 10.1371/journal.pone.0276716 (PMC9621403; doi:10.1371/journal.pone.0276716)
Supplement: S5 Table — (DOCX) [file pone.0276716.s006.docx]

**S6 Table. Factors influencing foot orthoses issue and review processes and magnitude of their influence relative to the practitioner’s years of clinical experience.**

|  | **All** |  | **< 1 year** |  | **1 - 5 years** |  | **6 - 10 years** |  | **11 - 15 years** |  | **> 15 years** |
| --- | --- | --- | --- | --- | --- | --- | --- | --- | --- | --- | --- |
|  | *n (%)* |  | *n (%)* |  | *n (%)* |  | *n (%)* |  | *n (%)* |  | *n (%)* |
| *Appointment availability* | | | | | | | | | | | |
| Minimally or not influential | 111 (53) |  | 6 (40) |  | 27 (41) |  | 23 (59) |  | 17 (55) |  | 38 (63) |
| Moderately influential | 69 (33) |  | 5 (33) |  | 25 (38) |  | 11 (28) |  | 11 (35) |  | 17 (28) |
| Highly influential | 31 (15) |  | 4 (27) |  | 14 (21) |  | 5 (13) |  | 3 (10) |  | 5 (8) |
| **Total** | **211** |  | **15** |  | **66** |  | **39** |  | **31** |  | **60** |
| *Clinic protocols / Employer preferences* | | | | | | | | | | | |
| Minimally or not influential | 96 (45) |  | 3 (20) |  | 28 (42) |  | 21 (54) |  | 16 (52) |  | 28 (47) |
| Moderately influential | 65 (31) |  | 5 (33) |  | 26 (39) |  | 11 (28) |  | 6 (19) |  | 17 (28) |
| Highly influential | 50 (24) |  | 7 (47) |  | 12 (18) |  | 7 (18) |  | 9 (29) |  | 15 (25) |
| **Total** | **211** |  | **15** |  | **66** |  | **39** |  | **31** |  | **60** |
| *Laboratory based factors (e.g. manufacture and delivery times)* | | | | | | | | | | | |
| Minimally or not influential | 77 (36) |  | 5 (33) |  | 20 (30) |  | 13 (33) |  | 12 (39) |  | 27 (45) |
| Moderately influential | 78 (37) |  | 4 (27) |  | 25 (38) |  | 17 (44) |  | 12 (39) |  | 20 (33) |
| Highly influential | 56 (27) |  | 6 (40) |  | 21 (32) |  | 9 (23) |  | 7 (23) |  | 13 (22) |
| **Total** | **211** |  | **15** |  | **66** |  | **39** |  | **31** |  | **60** |
| *Patient preferences* | | | | | | | | | | | |
| Minimally or not influential | 23 (11) |  | 1 (7) |  | 7 (11) |  | 3 (8) |  | 4 (13) |  | 8 (13) |
| Moderately influential | 112 (53) |  | 5 (33) |  | 34 (52) |  | 22 (56) |  | 18 (58) |  | 33 (55) |
| Highly influential | 75 (36) |  | 9 (60) |  | 24 (37) |  | 14 (36) |  | 9 (29) |  | 19 (32) |
| **Total** | **210** |  | **15** |  | **65** |  | **39** |  | **31** |  | **60** |
| *Professional judgement* | | | | | | | | | | | |
| Minimally or not influential | 9 (4) |  | 1 (7) |  | 2 (3) |  | 1 (3) |  | 2 (6) |  | 3 (5) |
| Moderately influential | 38 (18) |  | 2 (13) |  | 17 (25) |  | 4 (10) |  | 6 (19) |  | 9 (15) |
| Highly influential | 164 (78) |  | 12 (80) |  | 48 (72) |  | 34 (87) |  | 23 (74) |  | 47 (80) |
| **Total** | **211** |  | **15** |  | **67** |  | **39** |  | **31** |  | **59** |

*n* number of respondents in each category, % percentage of respondents in each category proportional to the total number of respondents with equivalent years of practice experience.
