# Supplementary material for: A cross-sectional analysis of podiatrist-initiated review processes after issuing prescribed foot orthoses
Source: PLoS One. 2022 Oct 31;17(10):e0276716. doi: 10.1371/journal.pone.0276716 (PMC9621403; doi:10.1371/journal.pone.0276716)
Supplement: Attachment — Submitted filename: Response to Reviewers.docx [file pone.0276716.s007.docx]

Dear Dr Carroll and the assigned Reviewers,

Please find below an itemised explanation of our responses to the feedback provided.

Thank you again for your consideration of our manuscript.

Kind regards,

Luke Donnan

**Academic editor**

| 1. Please ensure that your manuscript meets PLOS ONE's style requirements, including those for file naming. The PLOS ONE style templates can be found at  <https://journals.plos.org/plosone/s/file?id=wjVg/PLOSOne_formatting_sample_main_body.pdf> and  <https://journals.plos.org/plosone/s/file?id=ba62/PLOSOne_formatting_sample_title_authors_affiliations.pdf> | Title page has been adjusted to include corresponding author initials after the contact email address.  Figures, tables and supporting information documents are now correctly labelled.  Figures, tables and supporting information documents are now correctly acknowledged within the text.  Newly created figures are presented in .tif format. |
| --- | --- |
| 2. Please provide additional details regarding participant consent. In the ethics statement in the Methods and online submission information, please ensure that you have specified what type you obtained (for instance, written or verbal, and if verbal, how it was documented and witnessed). If your study included minors, state whether you obtained consent from parents or guardians. If the need for consent was waived by the ethics committee, please include this information.  Once you have amended this/these statement(s) in the Methods section of the manuscript, please add the same text to the “Ethics Statement” field of the submission form (via “Edit Submission”).  For additional information about PLOS ONE ethical requirements for human subjects research, please refer to <http://journals.plos.org/plosone/s/submission-guidelines#loc-human-subjects-research>. | Original wording:  *“The initial page of the online questionnaire provided information pertaining to the study, and consent was implied when a participant proceeded past the information page to the first question.”*  Revised wording:  *“The initial page of the online questionnaire included a participant information sheet and a consent form. If participants agreed to the terms outlined in the participant information sheet, consent was given by clicking ‘Next’ and proceeding to the survey questions.*  This modification has been applied to both the manuscript and to the ‘Ethics Statement’. |
| 3. Thank you for stating the following in the Acknowledgments Section of your manuscript:  "The authors would like to acknowledge Gail Fuller from the Spatial Data Analysis Network (SPAN) at Charles Sturt University for her assistance in preparing and administering the online questionnaire."  We note that you have provided funding information that is not currently declared in your Funding Statement. However, funding information should not appear in the Acknowledgments section or other areas of your manuscript. We will only publish funding information present in the Funding Statement section of the online submission form.  Please remove any funding-related text from the manuscript and let us know how you would like to update your Funding Statement. Currently, your Funding Statement reads as follows:  "We acknowledge financial support provided by Charles Sturt University. The funders had no role in study design, data collection and analysis, decision to publish, or preparation of the manuscript."  Please include your amended statements within your cover letter; we will change the online submission form on your behalf. | Thank you for these suggestions, although the authors may require further instruction. I assume our acknowledgement of Gail Fuller’s contribution in the Acknowledgements section is suitable.  The editor has queried the provision of funding information that was not in the Funding Statement. To our knowledge the only mention of funding information was in the ‘Financial Disclosure’ section which is separate to the manuscript text. Is it possible to be directed toward the other reference(s) to financial support that need modification? |
| 4. Please include captions for your Supporting Information files at the end of your manuscript, and update any in-text citations to match accordingly. Please see our Supporting Information guidelines for more information: <http://journals.plos.org/plosone/s/supporting-information>. | A Supporting Information section has been added at the end of the document. Labels and titles have been provided for all documents. Presentation of links to Supporting Information files within the text have also been adjusted to meet the journal requirements. |

**Reviewer 1**

| Thank you for the opportunity to review the manuscript titled ‘A cross-sectional analysis of podiatrist-initiated review processes after issuing prescribed foot orthoses’. While it fits the journal of submission, I would have thought a podiatry-focused journal may have been the target audience. However, whilst it is a relatively niche topic, the general principle related to duty of care, standardised practices and client centre management should have broad appeal. However again, given the journal audience, the rationale and presentation of outcomes of this survey could be stronger to enhance readership. The authors have covered the podiatry literature well but have stayed very podiatry-focused throughout. | We do agree with Reviewer 1 in that the premise of this manuscript may be suited to a podiatry-focused journal. We are also mindful that examples exist whereby podiatry specific content has been published in PLOS ONE:  *Banwell, H. A., Causby, R. S., Crozier, A. J., Nettle, B., & Murray, C. (2021). An exploration of the use of 3D printed foot models and simulated foot lesions to supplement scalpel skill training in undergraduate podiatry students: A multiple method study. PloS One, 16(12), e0261389–e0261389.* [*https://doi.org/10.1371/journal.pone.0261389*](https://doi.org/10.1371/journal.pone.0261389)  *Cockayne, S., Adamson, J., Clarke, A., Corbacho, B., Fairhurst, C., Green, L., Hewitt, C. E., Hicks, K., Kenan, A.-M., Lamb, S. E., McIntosh, C., Menz, H. B., Redmond, A. C., Richardson, Z., Rodgers, S., Vernon, W., Watson, J., & Torgerson, D. J. (2017). Cohort randomised controlled trial of a multifaceted podiatry intervention for the prevention of falls in older people (The REFORM Trial). PloS One, 12(1), e0168712–e0168712. https://doi.org/10.1371/journal.pone.0168712*    We would hope the content is deemed suitable for PLOS ONE and doesn’t hamper our prospects of publication. |
| --- | --- |
| Perhaps identifying concepts such as ‘there is very little evidence on what FOs do, therefore the need to monitor their effect on clients is paramount’ – highlighting duty of care or alike may offer depth of premise as well as identifying that standards of care/guidelines need to be based on best practice principles and, in an absence of those, at least having an understanding of what is happening is a start. The argument for review times may also be strengthened by comparing to other professions – is there literature or guidelines for orthotists? Optometrists or any other profession that dispenses medical devices? | It is the preference of the authors that we maintain the focus of manuscript. While we do acknowledge the concerns raised by Reviewer 1, the authors are mindful that bringing in abstract concepts may dilute the messaging of the manuscript.  We did take on board the suggestion regarding review times in other professions, particularly focusing on optical devices (as suggested), knee braces (from a physiotherapy or surgical perspective) and hand splints (from a physiotherapy or occupational therapy perspective). We were able to find some examples that examined the use of braces as a therapeutic modality but were unable to find resources that examined the review periods associated with those devices. |
| The writing style and the development of the manuscript has been well done. The authors should be commended on their efforts. | The authors are very appreciative of this feedback. |
| 1. Please clarify ‘consent was implied’ – was consent gained specifically? | A similar query was raised by the Academic Editor. To address this concern the following change has been made:  Original wording:  *“The initial page of the online questionnaire provided information pertaining to the study, and consent was implied when a participant proceeded past the information page to the first question.”*  Revised wording:  *“The initial page of the online questionnaire included a participant information sheet and a consent form. If participants agreed to the terms outlined in the participant information sheet, consent was given by clicking ‘Next’ and proceeding to the survey questions.* |
| 2. Where you have declared that ‘A third qualified author (AH) and an expert in online…’ Line 72, it would be helpful to put some references of previous work to show this author’s expertise. | The ‘expert in online questionnaire development’ leads the Charles Sturt University Spatial Data Analysis Network (SPAN) and part of their remit is questionnaire design and survey implementation. This staff member has been acknowledged in numerous publications and other outputs but is typically not listed as an author. This does make it difficult to reference previous work. We have chosen to better reflect this staff members role with the following text:  Original wording:  *“A third qualified podiatrist (AH) and an expert in online questionnaire development evaluated the questions and questionnaire structure to establish face validity.”*  Revised wording:  *“To establish face validity of the questionnaire a third qualified podiatrist (AH) evaluated the questions to confirm accuracy from a clinical perspective. Further scrutiny was provided by a colleague, not from a podiatry background, whose role within the institution was to provide questionnaire design support. They evaluated the questionnaire to identify and amend confusing, poorly articulated, or leading questions.”* |
| 3. Also good to identify how face validity was reviewed? | Please see the response to item 2 (above). |
| 4. The reference for SurveyMonkey is needed (not the link to the platform itself) | The authors have modified both the wording and the way in which Survey Monkey were referenced.  Original wording:  *“The questionnaire was prepared using the Survey Monkey platform (*[*https://www.surveymonkey.com/*](https://www.surveymonkey.com/)*).”*  Revised wording:  *“All survey data were collected using the SurveyMonkey.com platform.”*  We used the following recent reference for guidance on how to present this information in a PLOS ONE publication:  Li, L., Sullivan, A., Musah, A., Stavrianaki, K., Wood, C. E., Baker, P., & Kostkova, P. (2022). To Zoom or not to Zoom: A longitudinal study of UK population’s activities during the COVID-19 pandemic. *PloS One*, *17*(7), e0270207–. <https://doi.org/10.1371/journal.pone.0270207> |
| 5. Recruitment strategies via social media could be simplified to ‘social media associated with the Australian Podiatry Association or private pages aimed at disseminating podiatry related information’ rather than naming BTG. | Thank you for this recommendation. The authors have chosen to accept this suggestion and present an adapted version as is noted below:  Original wording:  *“The authors targeted Australian podiatrists by using existing podiatry contact lists, posting on the ‘Podiatry – Bridge the Gap’ podiatrist only Facebook site, promotion through the Australian Podiatry Association social media and communication platforms, and requests for podiatry contacts to distribute the questionnaire among their podiatry colleagues.”*  Revised wording:  *“The authors targeted Australian podiatrists by using existing podiatry contact lists, social media and official communications initiated by the Australian Podiatry Association, and private social media pages aimed at disseminating podiatry related information.”* |
| 6. I would recommend including a participant table in the manuscript (rather than as an appendices). It would be very beneficial to identify if the responding cohort represents the profession (e.g. compare to APHRA data). | The authors agree with this suggestion and have included Appendix 2 within the manuscript (now Table 1). The labelling of subsequent table sand appendices have been adjusted accordingly.  While reviewing this section some minor errors were noted when reporting respondent demographic information. All errors were corrected and are identifiable in the track changes version of the updated document. |
| 7. As a suggestion, creating a table to display the main findings (including all participants regardless of experience) as your primary outcome alone with the impact of experience visually displayed via graphs might be. Having it all combined into the same tables means the reader needs to ‘seek’ results rather than have them displayed for them. A graph using a ‘stacked column’ approach would allow readers to see the impact of experience immediately and allow comparisons more readily. | The authors have accepted this suggestion.  We agree that the use of figures in place of tables will improve the readers ability to compare results between groups. For consistency of presentation, we have changed tables 1, 2, and 3 to figures 1, 2 and 3, respectively. To allow the reader to seek the nuance of the raw data we have included what were formerly labelled as tables 1 (now S4 - related to figure 1), 2 (now S5 - related to figure 2) and 3 (now S6 - related to figure 3) as appendices to support those figures. |

**Reviewer 2**

| Abstract: The statement on line 15 that "published information guiding the clinical use of foot orthoses is scarce" is broad and needs to be further clarified or supported. | The authors do agree that this statement is broad. We have modified that sentence to reference the absence of clinical guidelines to direct orthotic prescription and review procedures.  Original wording:  *“As published information guiding the clinical use of foot orthoses is scarce, the aim of this study is to profile the review processes used by practicing podiatrists after issuing an orthotic device.”*  Revised wording:  *“In the absence of published guidelines directing the clinical use of foot orthoses, the aim of this study is to profile the review processes used by practicing podiatrists after issuing an orthotic device.”*  For Review 2’s benefit, we would also like to note that further qualification of this position has been provided within the Introduction section:  *“Recent systematic reviews indicate the efficacy of FO when treating foot pathology is inconsistent (5-7). While these publications do inform the clinical application of FO, each author contextualised their findings by acknowledging the variability that exists in FO construction and characteristics when comparing studies (5-7). Much like FO prescription habits, the review processes that follow an initial FO fitting are shown to vary between practitioners (8). This variation may reflect differing foci of the practitioner (8), potentially highlighting a practitioner interest in either symptom relief (4, 9-13) or biomechanical response (13, 14).”* |
| --- | --- |
| I would suggest reviewing the way that numbers are presented throughout the manuscript as there is some inconsistency. For example on line 26, thirty-two percent should be represented as 32%. | Thank you for identifying this error. The following change has been made:  Original wording:  *“A second review consultation was standard practice for thirty-two percent (n = 68) or respondents, and were typically scheduled either two (23%, n =12) or four (38%, n =20) weeks after the initial review consultation.”*  Revised wording:  *“A second review consultation was standard practice for 32% (n = 68) or respondents, and were typically scheduled either two (23%, n =12) or four (38%, n =20) weeks after the initial review consultation.”*  The remainder of the document has been checked and they use of words to identify percentage values is now limited to examples where that number is the first word in a sentence. |
| Introduction: Is the evidence to support the use of orthoses for back disorders strong enough to include it here? Similarly you mention that orthoses are believed to influence the function of the pelvis and thorax, is this supported by the evidence. The reference included on this statement by Cambron et al did not specifically investigate or discuss pelvic or thoracic biomechanics. | Thank you for identifying these items. We do agree with the concerns of Reviewer 2 regarding the strength of evidence to support the use of orthoses for the treatment of back disorders. The original sentence from our manuscript reads as follows:  *“Foot orthoses (FO) are commonly considered for the treatment and prevention of foot, lower limb and back disorders (1), typically with the intention of modifying loads on anatomical structures (2).”*  Our reference to back disorders emanates from the following sentences in the Cambron text:  *“Podiatrists have connected the use of foot orthoses for the*  *relief of LBP with the thought that back pain may be related to a disruption in the kinetic chain (3-5). However, it was not until the last decade that other investigators have begun to study the effect of foot function on the kinematics of the knee, hip, pelvis, and thorax (6-10).”*  To soften the language and confirm this is more of a commentary on practice rather than evidence, we have opted for the following revision:  *“Foot orthoses (FO) are considered by some practitioners for the treatment and prevention of foot, lower limb and back disorders (1), typically with the intention of modifying loads on anatomical structures (2).”*  With regard to the query about the pelvis and thorax, the sentence in our original manuscript reads as follows:  *“The use of FO as a mechanical intervention is believed to influence symptoms by changing function of the foot, leg, hip, pelvis and thorax (1, 4), although the precise way in which FO impact these structures is still not fully understood (2).”*  The Cambron reference had acknowledged that manipulation of foot position can impact pelvis and thorax position, however, you are correct to identify that Cambron was not the primary source of the information. Further investigation has also noted that the articles cited by Cambron that related to pelvis and thorax position (Pinto et al., 2008; Tateuchi et al., 2011) used protocols that simply manipulated calcaneus position.  To address this inaccuracy the authors have reworded the sentence in question to better reflect the evidence available and included Pinto and Tateuchi as primary sources of information:  *“Manipulation of foot position as a mechanical intervention is believed to influence symptoms by changing function of the foot, leg, hip, pelvis and thorax (1, 4-6), although the precise way in which these structures respond is still not fully understood (2).”* |
| Methods: You may benefit from clarification regarding the number of questions and specifically those questions that were omitted from this manuscript. I would suggest elaborating on what questions were excluded and why? Whilst I can understand your intention to focus this manuscript on orthotic review processes, that could have been a section of a more comprehensive review that may have provided further context for the findings. I can see in the survey within your appendix that questions which were not included relate to things like clinical assessment of orthotic suitability, patient education regarding wearing in the orthotic, adjunct interventions. These are all interesting questions which I believe would fit well into this manuscript. I do not see the benefit in removing these questions from this manuscript, I think the interest and clinical value of this study would be greatly improved if all questions were included. | Thanks for the comments and we do appreciate this does warrant further explanation. The authors have planned to disseminate the findings from this project in two parts. This manuscript is part one of two and relates to the review processes adopted by clinicians. Part two of two will focus on the clinical interaction component when issuing foot orthoses (assessments performed, education provided, adjunct therapies). The decision to present two manuscripts in shorter formats is intended to make the information focused and more easily understood for clinicians.  We had also hoped that provision of the full questionnaire (Appendix 1) and comments highlighting that only a portion of the project is being presented would ensure our process is transparent.  Line 101 *“As the purpose of this publication is to evaluate foot orthosis review procedures, results relating to 10 of the 25 questions from the questionnaire have been analysed”* |
| Results: You said that 238 registered podiatrists accepted the invitation. Do you have any way of confirming that they were registered? It may be better to just say podiatrists. | The authors have accepted this suggestion.  Original wording:  *“A total of 238 registered podiatrists accepted the invitation to complete the questionnaire.”*  Revised wording:  *“A total of 238 podiatrists accepted the invitation to complete the questionnaire.”* |
| When reporting the volume of prescriptions I would suggest reordering these from less than one pair, one to three pairs, four to six pairs etc. I can see that it is currently listed in order of response rate but feel that it would be easier to follow if ordered by number of orthotics. | The authors have accepted this suggestion.  Original wording:  *“When asked how many pairs of orthotics were prescribed in a standard week, most respondents reported one to three pairs (39%, n = 91), less than one pair (23%, n = 54) or four to six pairs (21%, n = 49). Nine percent of respondents prescribed between seven and nine pairs in a standard week (n = 20), and fewer prescribed 10 or more pairs (7%, n = 16).”*  Revised wording:  *“When asked how many pairs of orthotics were prescribed in a standard week, most respondents reported* *less than one pair (23%, n = 54), one to three pairs (39%, n = 91) or four to six pairs (21%, n = 49). Nine percent of respondents prescribed between seven and nine pairs in a standard week (n = 20), and fewer prescribed 10 or more pairs (7%, n = 16).”* |
| The total of people who prescribe polypropylene, EVA or carbon does not equal 100%. I understand that there are other materials considered such as PA11 and that these are included in your appendix. You may benefit from briefly listing these here. | The authors acknowledge the point being made, however, the way in which Question 4 was asked the value reported will not add up to 100%. The options provided in the questionnaire allowed respondents to choose ‘more than 50%’, ‘less than 50%’ or ‘none/rarely’ for each orthotic material. As the responses sit within a loose range (above or below 50%) and respondents may have selected either ‘less than 50%’ or ‘none/rarely’ for two of the three questions, the outcome of this question is intended to be a guide rather than being precise. |
| Table 2. The totals do not add up for the question of how many weeks after you schedule your second follow up. Eg the total people who do use a second follow up is 68 people, yet there are only 52 responses to how many weeks later the session is. Is this data missing? | Thank you for picking this up Reviewer 2. We have included an additional six rows which account for the missing data (now Fig 2 and S5 Table).  To ensure consistent results are reported throughout, four values reported in the ‘Scheduling of review consultations’ section were also updated. All corrections are identifiable in the track changes version of the updated document. |
| Discussion: Line 207-209. Studies assessing the efficacy of orthotic devices have used a broad range of follow up times. Perhaps reword this sentence so that it is not portrayed as an exhaustive list. | The authors have accepted this suggestion.  Original wording:  *“Studies assessing the efficacy of orthotic devices have used initial review periods of two (9, 15-17), four (11, 18) and six weeks (4, 12), typically with no justification as to why those time frames were chosen”*  Revised wording:  *“A sample of studies assessing the efficacy of orthotic devices have used initial review periods of two (9, 15-17), four (11, 18) and six weeks (4, 12), typically with no justification as to why those time frames were chosen”* |
| Given the reliance on "professional judgement" that was found, it would be interesting to know what informed these judgements. For example, do these relate to pathology/diagnosis, biomechanics of the patient or objective, or other? | The authors do agree that this would be an area of interest for future research. Unfortunately, the question did not seek additional information from the participants to inform such commentary. |
| Line 247. This sentence would read better if begun with When Cambron et al compared the use of.... | The authors have accepted this suggestion.  Original wording:  *“When comparing the use of 1) no-intervention, 2) orthotic intervention or 3) orthotics plus chiropractic intervention, on lower back pain, all groups reported significant reductions in pain when reviewed at 12 months and no differences were observed between groups (4).”*  Revised wording:  *“When Cambron et al. (2017) compared the use of 1) no-intervention, 2) orthotic intervention or 3) orthotics plus chiropractic intervention, on lower back pain, all groups reported significant reductions in pain when reviewed at 12 months and no differences were observed between groups (4).”* |
| You had representation from 18 universities in the study, did this factor into the analysis, and if so, was a difference noted? | We too were interested in this aspect of analysis and expected the variation would have related to instruction provided during tertiary studies. This was considered during analysis and no such relationships were identified. This does not mean that a relationship does not exist, but with such small representation from many of the institutions it is not possible to confirm based upon this data set. |
| Line 303. You have stated in the limitations that these results may not be generalisable so should consider adjusting this sentence to read "this study has identified variation in the orthotic review processes amongst podiatrists who responded" or something similar. | The authors have accepted this suggestion.  Original wording:  *“This study has identified variation in the orthotic review processes used by practicing podiatrists, although it is not yet clear why practitioners adopt such varied approaches.”*  Revised wording:  *“This study has identified variation in the orthotic review processes among those podiatrists who responded, although it is not yet clear why practitioners adopt such varied approaches.”* |
| I would be interested to know how you think these results will influence practice or future research? Maybe add a short section regarding future directions and research significance. | With regard to future directions, the authors did note (Line 330):  “Further investigation may be beneficial to understand why practitioners have adopted these approaches and if the review process has any impact on patient outcomes.”  There were also comments relating to the relevance of the findings. Most importantly, this study identified an area of practice that is highly subjective and not directed by evidence (Line 326):  *“This study has identified variation in the orthotic review processes used by practicing podiatrists, although it is not yet clear why practitioners adopt such varied approaches.”*  …and (Line 332):  *“The findings of this study may also provide insights into the clinical decision making of experienced practitioners prescribing orthotic devices, and the factors that influence novice practitioners still developing their clinical procedures.”*  However, as emerging researchers we have opted to not be overly controversial with our comments directed at our profession. If Reviewer 2 believes stronger language is required, we would consider further adjustments. |

**Adherence to CHERRIES reporting requirements**

| **Item category** | **Checklist item** | **Location (on track changes version)** |
| --- | --- | --- |
| Design | Describe survey design | Line 116 |
| IRB (Institutional Review Board) | IRB approval | Line 65 |
|  | Informed consent | Line 67 |
|  | Data protection | Line 138 |
| Development and pre-testing | Development and testing | Development: Line 74, Testing: Line 92 |
| Recruitment process | Open survey vs closed survey | Line 82 |
|  | Contact mode | Line 118 |
|  | Advertising the survey | Line 118 |
| Survey administration | Web/E-mail | Line 83 |
|  | Context | Line 83 |
|  | Mandatory/voluntary | Line 125 |
|  | Incentives | Line 85 |
|  | Time/Date | Line 137 |
|  | Randomisation of items | Line 100 |
|  | Adaptive questioning | Line 136 |
|  | Number of items | Line 100 |
|  | Number of screens | Line 101 |
|  | Completeness check | Line 102 |
|  | Review step | Line 104 |
| Response rates | Unique site visitor | Not used in this study. |
|  | View rate | Not used in this study. |
|  | Participation rate | Line 157 |
|  | Completion rate | Line 158 |
| Prevent multiple entries from the same user | Cookie used | Not used in this study. |
|  | IP check | Line 161 |
|  | Log file analysis | Not used in this study. |
|  | Registration | Not used in this study. |
| Analysis | Handling of incomplete questionnaires | Line 162 |
|  | Questionnaires with atypical timestamp | Not used in this study. |
|  | Statistical correction | Not relevant to this study as authors reported only descriptive statistics. |
